# A Case of Congenital Disorder of Glycosylation Ia Presented with Recurrent Pericardial Effusion

**Published:** 2014-07-19

**Authors:** Sedat Işıkay, Osman Başpınar, Kutluhan Yılmaz

**Affiliations:** 1 Department of Pediatric Neurology; 2 Department of Pediatric Cardiology, Gaziantep Children’s Hospital, Gaziantep; 3 Department of Pediatric Neurology, Medeniyet University, İstanbul, Turkey

**Keywords:** Congenital Disorder of Glycosylation Ia, Pericardial Effusion, Inborn Error of Metabolism, Dysmorphia

## Abstract

***Background:*** Inherited deficiency of phosophomannomutase (PMM2) causes a human glycosylation disorder known as Congenital Disorder of Glycosylation Ia.

***Case Presentation:*** Herein, we describe a case of congenital disorder of glycosylation Ia, presented with recurrent pericardial effusion and unusual findings of inverted nipples, fat pads, reduced deep-tendon reflexes and multisystem involvement.

***Conclusion:*** Congenital Disorder of Glycosylation Ia should be considered in children with developmental delay, those with multi-system disease involving neurologic, gastrointestinal, ophthalmologic, cardiac or endocrine systems. On the other hand, severe cardiac involvement may also be a feature of Congenital Disorder of Glycosylation Ia and diagnosed patients should also be evaluated in this respect.

## Introduction

Inherited deficiency of phosophomannomutase (PMM2) causes a human glycosylation disorder known as Congenital Disorder of Glycosylation Ia (CDG-Ia)^[^^1^^]^. Patients have a broad and variable clinical picture that affects nearly all systems leading to failure to thrive, hypotonia, variable developmental delay, dysmorphia, and unfortunately with up to 20% mortality in the first 5 years of life due to organ failure and/or severe infections^[^^2^^]^. Herein, we will describe a case of CDG-Ia, presented with recurrent pericardial effusion and unusual finding of inverted nipples, fat pads, reduced deep-tendon reflexes and multisystem involvement.

## Case Presentation

**Fig. 1:**
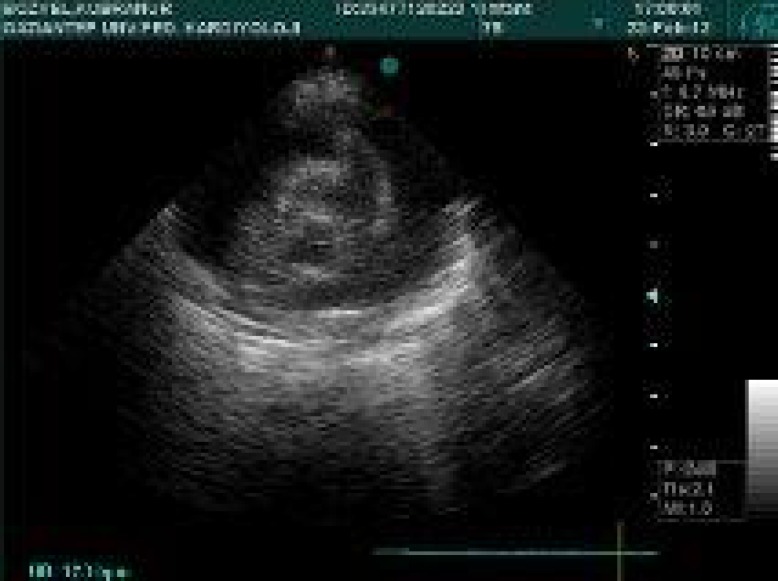
Pericardial effusion and cardiac tamponade with right ventricular pressure findings

A 7-month-old girl was referred to the pediatric clinic with developmental delay and failure to thrive. Her family history and antenatal course were unremarkable. At presentation, she was noted to have central hypotonia, hyporeflexia, and dysmorphic features including high arched palate, narrow palpebral fissures and inverted nips. There were prominent fat pads on her supragluteal regions and global developmental delay. At admission, her respiratory and myocardial functions were deteriorated and there was an escalating pericardial effusion and we had to perform pericardiocentesis for two times (Fig. 1) and as it did not resolve, a pericardial window had to be opened in order to drain the effusion and a pericardial biopsy was also performed.

**Fig. 2:**
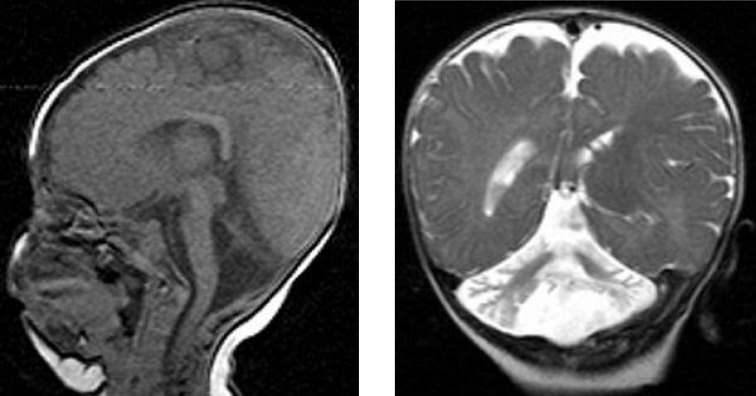
Coronal T2 (A) and sagittal T1 (B) magnetic resonance images showing cerebellar vermis hypoplasia and a generalized reduction in myelination

The pericardial effusion was sero-sanguinolent and the biopsy revealed mild inflammation and fibrous thickening of peritoneum. Investigations for a metabolic etiology at this time confirmed normal levels of urine amino acids and organic acids. Blood measurements including levels of plasma amino acids, biotinidase, ammonia, vitamin B_12_ and lactate were all normal. There was no documented episode of hypoglycemia, presence of urinary ketones and/or metabolic acidosis. TORCH panel involving testing for antibodies to *Toxoplasma gondii*, rubella, cytomegalovirus and herpes simplex virus was negative. Abdominal ultrasound revealed hyperechogenicity of the renal parenchyma. Magnetic resonance imaging of brain revealed a cerebellar vermis hypoplasia and a generalized reduction in myelination (Fig. 2). The child was felt to have a phenotype classic of the CDG-Ia. Isoelectric focusing of transferrin showed a pattern consistent with CDG-Ia. Genetic analysis revealed that she was homozygote c.[385G>A]+[385G>A] for pathogenic mutation in phosphomannomutase 2 *(PMM2) *gene*, *consistent with a diagnosis of CDG-Ia. In 14^th^ month of follow-up, the pericardial effusion did not recur; the general condition is good with normal heart functions.

## Discussion

The differential diagnosis of developmental delay is broad. However, in a patient like ours who has many systems affected, a multisystem disease should be suspected. In this case, the phenotype matched that of CDG-Ia, and results of specific functional and genetic tests confirmed the diagnosis.

Congenital Disorder of Glycosylation refers to an inherited group of metabolic disorders due to defects in post-translational modification of glycosylated peptides. Over 500 genes are involved in glycosylation process, and half of the body’s proteins are glycoproteins. Thus, disorders of glycosylation encompass many diseases with diverse presentations, and they may involve one or many systems. CDGs are believed to affect people of any ethnic background, and the estimated prevalence is 1.5 per 100,000 population^[3]^. CDG-Ia is the most common type of congenital glycosylation disorder reported in childhood. Patients with CDG-Ia may have neurologic disease solely or neurologic disease and multisystem involvement. Isoelectric focusing of transferrin is a screening test for CDG. It cannot reliably identify the subtype of CDG, and not all types can be detected this way. This blood test relies on a difference in the number of sialic acid side chains on the glycoprotein, with the resulting change in the net charge of the glycoprotein affecting the migration patterns on the gel. In CDG-Ia, mutations of the *PMM2* gene on chromosome 16p13 result in a deficiency of the PMM2 enzyme^[^^3^^]^. This enzyme converts mannose 6-phosphate to mannose 1-phosphate, and a deficiency results in abnormal glycosylation. PMM is widely expressed in the body, which explains the multi-system disease associated with this condition. The disorder is confirmed by identification of gene mutations or by decreased PMM enzyme activity in fibroblast cultures. CDG-Ia is a very broad spectrum of disease ranging from no involvement with normal development to significant neurological impairment with multisystem involvement^[^^4^^]^. Cardiac involvement in CDG-Ia has been reported before. Clayton et al described a patient with respiratory distress and a murmur with episodes of arterial oxygen desaturation and diagnosed with neonatal-onset CDG. A new feature of the disease was severe hypertrophic cardiomyopathy^[^^5^^]^. Rudaks et al^[^^6^^]^ recently reported two female siblings with CDG-Ia and cardiomyopathy who died from hyperthrophic cardiomyopathy in the first 2 months of life and they emphasized the importance of early cardiac assessment in all patients with CDG-Ia. On the other hand, Petersen et al^[^^7^^]^ reported 5 cases with CDG and all 5 children were seen during their first year of life with failure to thrive, psychomotor retardation, inverted nipples, and pericardial effusion. Then, Chang et al^[^^8^^]^ reported the case of an 8-month-old male infant who presented in the neonatal period with failure to thrive, bilateral pleural and pericardial effusion, and hepatic insufficiency. Truin et al^[^^9^^]^ described the three children carrying severe *PMM2 *mutations who developed life-threatening extra vascular fluid accumulation. Both pericardial and abdominal fluid accumulations were reported in those cases and in 2 of them severe extravascular fluid accumulation progressed to decompensation and death^[^^9^^]^. Abnormal glycosylation of cell surface proteins may result in disequilibrium of normal fluid balance and protein transport through the pericardial and peritoneal membranes and may cause this life-threatening complication of CDG syndrome.

## Conclusion

In the case reported here, the patient’s non-specific but unusual findings of recurrent pericardial effusion, inverted nipples, fat pads, reduced deep-tendon reflexes and multisystem involvement pointed us toward a diagnosis other than cerebral palsy and the cerebellar vermis hypoplasia and a generalized reduction in myelination reinforced the early clinical suspicion of a PMM2-CDG disease. CDG-Ia should be considered in children with developmental delay, those with multi-system disease involving neurologic, gastrointestinal or cardiac systems. On the other hand, severe cardiac involvement may also be a feature of CDG-Ia and diagnosed patients should also be evaluated in this respect.
